# Comparison of Different Methods to Determine the DNA Sequence Preference of Ionising Radiation-Induced DNA Damage

**DOI:** 10.3390/genes11010008

**Published:** 2019-12-20

**Authors:** Vincent Murray, Megan E. Hardie, Shweta D. Gautam

**Affiliations:** School of Biotechnology and Biomolecular Sciences, University of New South Wales, Sydney, NSW 2052, Australia; megan@unswalumni.com (M.E.H.); shweta.dutta08@gmail.com (S.D.G.)

**Keywords:** DNA damage, DNA sequence preference, fluorescently end-labelled DNA, gamma radiation, genome-wide sequencing, polymerase stop assay

## Abstract

Ionising radiation (IR) is known to induce a wide variety of lesions in DNA. In this review, we compared three different techniques that examined the DNA sequence preference of IR-induced DNA damage at nucleotide resolution. These three techniques were: the linear amplification/polymerase stop assay, the end-labelling procedure, and Illumina next-generation genome-wide sequencing. The DNA sequence preference of IR-induced DNA damage was compared in purified DNA sequences including human genomic DNA. It was found that the DNA sequence preference of IR-induced DNA damage identified by the end-labelling procedure (that mainly detected single-strand breaks) and Illumina next-generation genome-wide sequencing (that mainly detected double-strand breaks) was at C nucleotides, while the linear amplification/polymerase stop assay (that mainly detected base damage) was at G nucleotides. A consensus sequence at the IR-induced DNA damage was found to be 5′-AGGC*C for the end-labelling technique, 5′-GGC*MH (where * is the cleavage site, M is A or C, H is any nucleotide except G) for the genome-wide technique, and 5′-GG* for the linear amplification/polymerase stop procedure. These three different approaches are important because they provide a deeper insight into the mechanism of action of IR-induced DNA damage.

## 1. Introduction

### 1.1. DNA Damage Induced by Ionising Radiation

Ionising radiation (IR) is known to cause mutations and cancer [1,2,3,4]; however, it is also clinically utilised to treat cancer [5,6,7]. There are a large number of different DNA lesions produced by IR. These include: double-strand breaks (DSBs), single-strand breaks (SSBs), abasic (apurinic/apyrimidinic) sites, intramolecular crosslinks, oxidised bases and sugars, and protein–DNA crosslinks [6,8,9,10,11,12,13,14,15,16,17,18,19,20,21,22,23]. These lesions have been quantified and it has been estimated that IR induces approximately 20–40 DSBs, 1000 SSBs, and 1000–1300 base lesions/cell/Gy [24,25,26,27,28,29,30,31]. It is likely that IR induces several hundred different lesions in DNA [12,20].

IR induces DNA damage through two main mechanisms: direct and indirect effects. The direct effects occur without an intermediary, while the indirect effects occur via radiolysis of solvent molecules. The radiolysis of water produces a number of free radicals that can react with and damage DNA. The major free radicals produced by radiolysis are hydroxyl radicals, hydrogen radicals, superoxide radicals, hydrogen peroxide and solvated electrons [15]. The only free radical that can produce substantial damage to DNA is the hydroxyl radical [32]. The hydroxyl radical can cause both SSBs and DSBs via reaction with the deoxyribose sugar of DNA [15,33,34]. IR also induces DNA damage to the bases in DNA. The purine bases are damaged to a greater extent than the pyrimidines [28,35,36]. The major purine lesions induced by IR are: 8-oxo-7,8-dihydroguanine (8-oxoG), 2,6-diamino-4-hydroxy-5-formamidopyrimidine (Fapy-G), 8-oxo-7,8-dihydro-2′-deoxyadenosine (8-oxoA) and 4,6-diamino-5-formamidopyrimidine (Fapy-A). The major pyrimidine lesions are the thymine glycols that are the cis- and trans-diasteromers of 5,6-dihydroxy-5,6-dihydrothymine (Thy-Gly) [37,38].

Apurinic/apyrimidinic (also known as abasic) sites are also produced by IR and can be identified by a number of enzymes [39,40,41,42,43,44,45,46]. These apurinic/apyrimidinic-specific enzymes can be used to detect the level of modified bases after IR treatment [33,34,47].

The IR-induced damage site can be several nucleotides from the initial lesion [48,49]. A transfer process can occur whereby an electron loss centre (hole) can move along the DNA until it reaches a site with the lowest ionisation energy which is usually the guanine base [50,51]. In DNA with GG and GGG sequences, the hole is located at the 5′-G in a run of consecutive guanine nucleotides [52,53,54,55,56,57,58,59].

It has been estimated that over 100 oxidatively generated DNA lesions and modifications are induced by IR [12]. A number of these lesions can be formed along the radiation track and thus can contribute to the complexity of IR-induced DNA damage.

### 1.2. Techniques to Determine the Sequence Preference of IR-Induced DNA Damage

**Figure 1:**
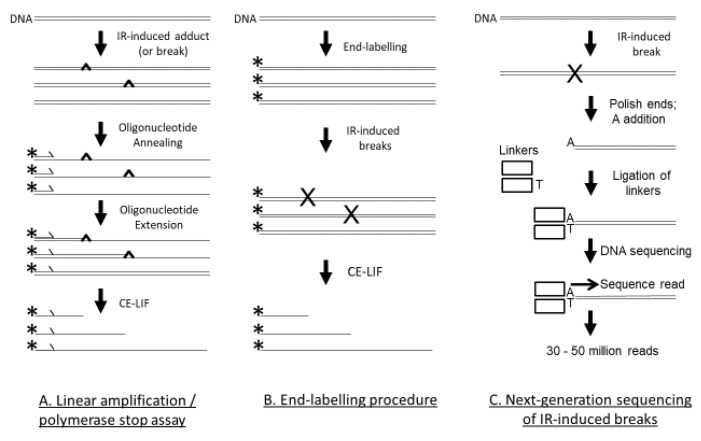
Three techniques that were used to assess IR-induced DNA damage. (**A**) With the LA/PS assay, after the double-stranded DNA is subjected to IR, the DNA is denatured and a fluorescently labelled oligonucleotide primer is annealed to the DNA. The labelled primer is extended by *Taq* DNA polymerase until terminated by a DNA adduct or break. Linear amplification is achieved by thermal cycling. The fluorescently labelled products are quantified by CE-LIF with reference to DNA sequencing reactions as size markers. (**B**) For the end-labelling technique, the DNA (usually a PCR product) is labelled at only one end—either the 5′ or 3′ end. The fluorescently labelled DNA is then treated with IR. The DNA breaks are quantified by CE-LIF with reference to DNA sequencing reactions as size markers. (**C**) With the Illumina next-generation genome-wide sequencing technique, genomic DNA is treated with IR. The DSBs are end-polished with a 3′–5′-exonuclease and a 5′–3′ DNA polymerase, followed by single A nucleotide addition, and then linker addition. This DNA is then added to the Illumina flowcell for DNA sequencing with the resultant production of 30–50 million reads. The cleavage site is at the 5′ end of the sequence read. The reads are mapped to the human genome and the DNA sequence at the cleavage sites is ascertained.

There are three techniques that have recently examined the DNA sequence preference of IR-induced DNA damage at nucleotide resolution using cutting-edge technology: the linear amplification/polymerase stop (LA/PS) assay [60], the end-labelling procedure [61], and Illumina next-generation genome-wide sequencing [62], as seen in Figure 1.

In the LA/PS assay, a labelled primer is extended by DNA polymerase until it encounters a DNA lesion that terminates chain extension, as seen in Figure 1. By use of DNA sequencing reactions as size standards that use the same labelled primer, the precise position of the DNA lesion in the DNA sequence can be determined. The LA/PS assay utilises capillary electrophoresis with laser-induced fluorescence (CE-LIF) detection that can accurately quantify IR-induced DNA lesions to the precise nucleotide. The LA/PS assay is capable of detecting DSBs, SSBs, abasic sites and base damage. It was concluded from a recent study [60] that the LA/PS assay mainly detected base damage induced by IR. G nucleotides were the main site of IR-induced DNA damage [60].

In the end-labelling procedure, a DNA sequence, usually a PCR product, is labelled at only one end (either the 5′ or 3′ end), as seen in Figure 1. After treatment with IR, any breaks in the DNA are detected by the CE-LIF procedure and can be accurately quantified to the exact nucleotide with reference to DNA sequencing reactions as size standards. The end-labelling procedure mainly detects SSBs. A recent study observed that C nucleotides were the main site of IR-induced DNA damage [61].

In the Illumina next-generation genome-wide sequencing technique, a whole genome can be treated with IR and the precise sites of cleavage can be determined, as seen in Figure 1. In this technique, double-stranded linkers are enzymically added at the cleavage site, placed on the Illumina flowcell for sequencing, and tens of millions of sequencing reads can be obtained. The 5′ end of the sequencing read is the cleavage site and after the sequencing read is mapped back to the human genome, the sequence at the cleavage site can be determined. By analysis of tens of millions of cleavage sites, an accurate picture of IR-induced cleavage in the entire human genome can be established. The Illumina next-generation genome-wide sequencing technique mainly detects DSBs. A recent study with the human genome found that C nucleotides were the main site of IR-induced DNA damage [62].

### 1.3. Aim

In this review, the aim was to compare the DNA sequence preference of IR-induced DNA damage using the three different techniques: the end-labelling procedure, the LA/PS assay, and genome-wide sequencing. It is important to use a number of techniques to examine IR-induced DNA damage because of the large number of different DNA lesions produced by IR.

## 2. Materials and Methods

The Mito 15 sequence [60,63] is a pUC19 plasmid that has a 246 bp human mitochondrial sequence inserted at the *Sma*I site. The inserted mitochondrial sequence corresponds to bp 11,851 to 12,097 in the hg19 human genome. The T7.GCGT.G10 sequence [60,61] is also based on pUC19 and consists of seven tandem repeats of a human telomeric sequence (5′-GGGTTA), a central 5′-GCGT repeating unit with adjacent sequence variants, and a sequence of ten consecutive G nucleotides. The J clone [60] contains a number of consecutive G nucleotides with different lengths. Purified human genomic DNA was utilised for the genome-wide studies [62].

The IR-induced DNA damage was generated by γ-irradiation from a sealed caesium-137 source that irradiated at a rate of 5.02 Gy/min at ambient atmosphere and temperature [60,61,62]. Experimental details of the three technique have been previously published for the LA/PS assay [60], the end-labelling technique [61] and the short-read Illumina next-generation genome-wide sequencing technique [62]. Purified DNA was utilised as the target for these three IR-induced DNA damage studies [60,61,62].

In the LA/PS assay [60], the purified DNA sequences were dissolved in H_2_O and irradiated with 50 Gy IR. The samples were then subjected to the LA/PS procedure where a fluorescently-labelled primer was extended by *Taq* DNA polymerase until inhibited by a DNA lesion. The LA/PS procedure utilised thermal cycling to linearly amplify the signal. The samples were analysed by CE-LIF with quantification by GeneMapper software [60,61,64,65,66]. For each DNA sequence examined, three independent experiments were performed. The precise sites of DNA damage were determined at nucleotide resolution with reference to dideoxy sequencing reactions.

With the end-labelling technique [61], PCR products were fluorescently labelled at either the 5′ or 3′ end, and the purified DNA was irradiated with 50 Gy IR in H_2_O. CE-LIF fragment analysis was performed and IR-induced DNA damage was quantified by GeneMapper software. The exact position of the DNA lesions was determined using DNA sequencing reactions as size standards. The 5′ and 3′ end data was combined to provide an unbiased DNA damage profile.

For the genome-wide sequencing technique [62], purified human DNA was dissolved in H_2_O and irradiated with 75 Gy IR. The DNA samples were processed with the TruSeq Nano preparative methodology where the ends were polished with DNA polymerase, followed 3′-A addition and linker ligation. After purification, the DNA samples were sequenced on an Illumina flow cell. The resultant 75 bp reads were mapped to the hg19 repeat masked human reference sequence using Bowtie software to determine the precise sites of the IR-induced DSBs. Further bioinformatic analysis was performed with Samtools and other visualisation tools.

In this paper, data was extracted from these three previous IR-induced DNA damage studies [60,61,62] to further analyse and compare the three techniques.

## 3. Results

### 3.1. Comparison of IR-Induced DNA Damage in a Human Mitochondrial Sequence

A human mitochondrial sequence was present in two different environments—human genomic DNA and a purified DNA sequence (Mito 15). The sequence preference of IR-induced DNA damage was compared using three different techniques: Illumina next-generation genome-wide sequencing (human genomic DNA), the LA/PS assay (Mito 15), and the end-labelling procedure (Mito 15).

**Figure 2:**
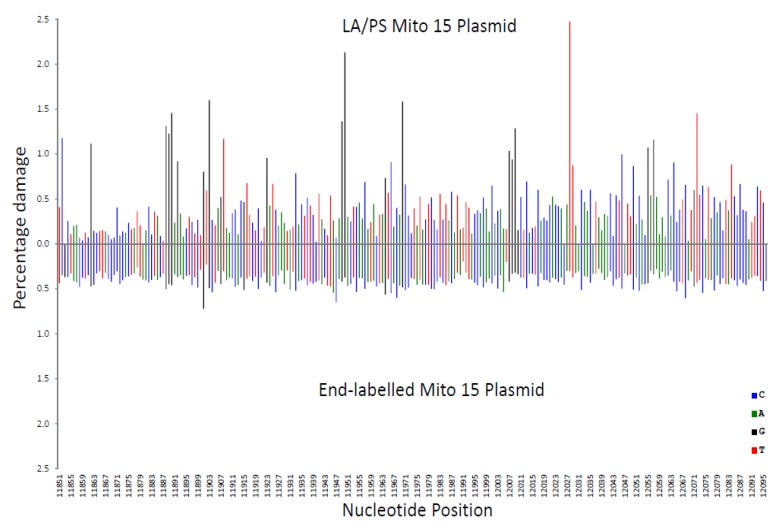
Comparison of the percentage damage intensity as assessed by the LA/PS assay and the end-labelling technique for a mitochondrial sequence. The IR-induced DNA damage intensities were normalised as a percentage of total damage within the mitochondrial region (from bp 11,851 to bp 12,096 in the human mitochondrial genome). The x-axis, linearly, displays the nucleotide position in the sequence. The y-axis represents the percentage damage intensity. The damage intensity peaks are green for A, blue for C, black for G and red for T. Note that the DNA polymerase approaches from the right of the figure (3’-end) with the LA/PS procedure. The end-labelling data is an average of 5’- and 3’-end labelling experiments. Top. LA/PS assay with the Mito 15 sequence. Bottom. End-labelling technique with the Mito 15 sequence.

In Figure 2, the LA/PS procedure was compared with the end-labelling procedure. It can be observed that over the analysed mitochondrial DNA sequence, the end-labelling procedure produced a relatively even pattern, while the LA/PS procedure produced a wider variation in percentage damage.

**Figure 3:**
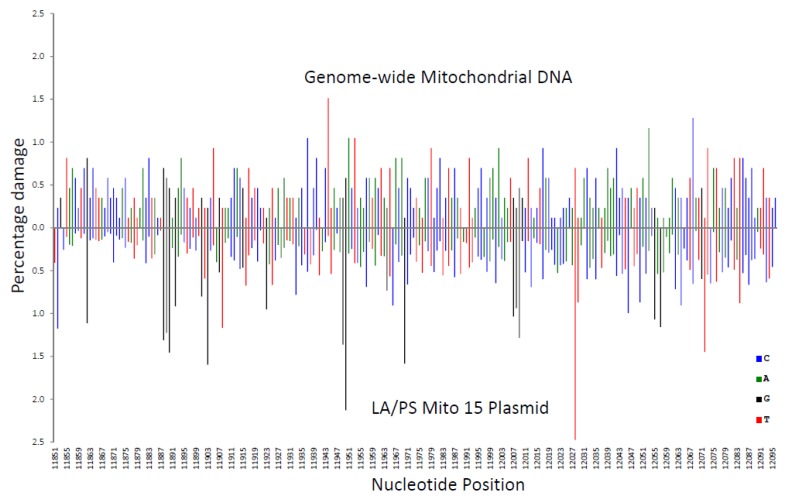
Comparison of the percentage damage intensity as assessed by the LA/PS assay and the genome-wide technique for a mitochondrial sequence. The IR-induced DNA damage intensities were normalised as a percentage of total damage within the mitochondrial region (from bp 11,851 to bp 12,096 in the human mitochondrial genome). The x-axis, linearly, displays the nucleotide position in the sequence. The y-axis represents the percentage damage intensity. The damage intensity peaks are green for A, blue for C, black for G and red for T. Note that the DNA polymerase approaches from the right of the figure (3’-end) with the LA/PS procedure. Top. Illumina next-generation genome-wide technique in purified human DNA with endonuclease IV treatment. Bottom. LA/PS assay with the Mito 15 sequence.

In Figure 3, the Illumina genome-wide procedure was compared with the LA/PS procedure. Across the analysed sequence, both procedures produced wide variations in percentage damage with peaks generally not coinciding at the same nucleotides.

**Figure 4:**
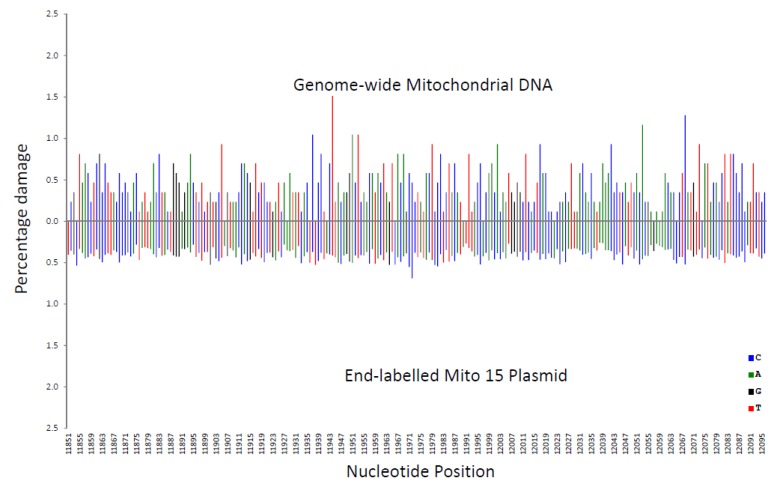
Comparison of the percentage damage intensity as assessed by the end-labelling technique and the genome-wide technique for a mitochondrial sequence. The IR-induced DNA damage intensities were normalised as a percentage of total damage within the mitochondrial region (from bp 11,851 to bp 12,096 in the human mitochondrial genome). The x-axis, linearly, displays the nucleotide position in the sequence. The y-axis represents the percentage damage intensity. The damage intensity peaks are green for A, blue for C, black for G and red for T. The end-labelling data is an average of 5’- and 3’-end labelling experiments. Top. Illumina next-generation genome-wide technique in purified human DNA with endonuclease IV treatment. Bottom. End-labelling technique with the Mito 15 sequence.

In Figure 4, the genome-wide procedure was compared with the end-labelling procedure. The genome-wide procedure produced greater variations in percentage damage compared with the more even pattern for the end-labelling procedure.

These variations in percentage damage were quantified by a standard deviation/average ratio. The standard deviation of the percentage damage divided by the average percentage damage was 0.65 for genome-wide, 0.87 for LA/PS, and 0.17 for the end-labelling procedure. These ratios confirmed the visual observations.

A correlation coefficient (R^2^ value) for each nucleotide was calculated for the three comparisons shown in Figure 2, Figure 3 and Figure 4. These R^2^ values were found to be 0.020 for end-labelling vs. LA/PS, 0.0011 for genome-wide vs. LA/PS, and 0.0068 for genome-wide vs. end-labelling. These R^2^ values indicated a very low correlation between the three procedures. The damage percentages at individual nucleotides were most similar for the end-labelling vs. LA/PS procedures, intermediate for the genome-wide vs. end-labelling procedures, and lowest for the genome-wide vs. LA/PS procedures.

### 3.2. The Sequence Preference of IR-Induced DNA Damage as Assessed by Three Different Techniques

**Figure 5:**
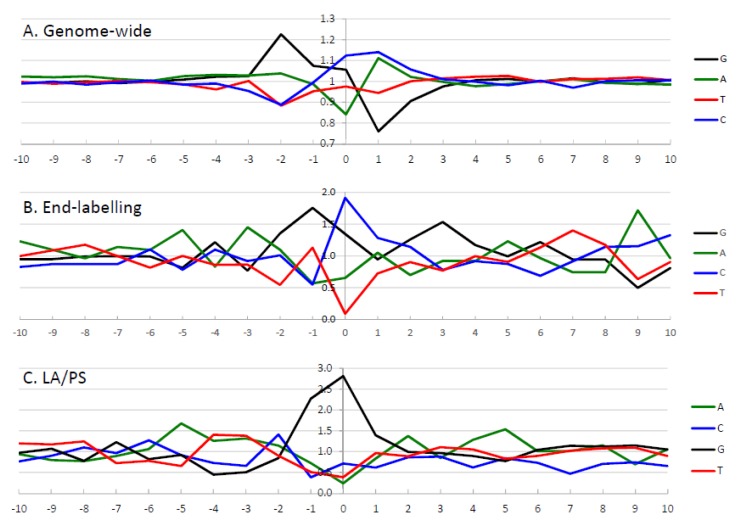
Frequency analysis of the highest intensity IR-induced DNA damage sites as assessed by the three different techniques. The frequency of occurrence of nucleotides at the highest intensity sites was calculated after adjustment for the nucleotide frequency in the DNA sequence of interest. The y-axis represents the ratio of observed/expected nucleotides. The x-axis represents the nucleotide position surrounding the damage site, from −10 to 10 bp, with the damage site indicated by zero. The calculated observed/expected nucleotide ratios are green for A, blue for C, black for G, and red for T. (**A**) Illumina next-generation genome-wide technique from the 50,000 highest intensity IR-induced cleavage sites in purified human DNA with endonuclease IV treatment [62]. (**B**) End-labelling technique with the 10% highest intensity IR-induced DNA damage sites from the combined Mito 15 and T7.GCGT.G10 (without telomeres) sequences [61]. (**C**) LA/PS assay with the 10% highest intensity IR-induced DNA damage sites from the combined Mito 15, T7.GCGT.G10 and the J clone sequences [60]. Parts of this Figure have been adapted from [60,62].

The frequency of nucleotides at IR-induced DNA damage sites is shown in Figure 5. For the genome-wide and end-labelling techniques, the predominant nucleotide at the damage site (position 0) was C; while for LA/PS it was G.

The impact of neighbouring nucleotides on the degree of IR-induced DNA damage was assessed by examining ten nucleotides on either side of the DNA damage site for the highest intensity sites. The observed/expected frequency ratio was determined, after correcting for the frequency of occurrence of nucleotides in the analysed sequence, from the “−10” position to the “10” position for the three techniques, as seen in Figure 5.

#### 3.2.1. The Genome-Wide Nucleotide Preference

For the genome-wide data, as seen in Figure 5A, the 50,000 highest intensity sites were examined to determine the influence of neighbouring sequences [62]. At the cleavage site (position “0”), C nucleotides were preferred, followed by G nucleotides. In the surrounding sequence, G nucleotides were preferred at the “−2” and “−1” positions, with the G nucleotide at “−2” having the most prominent observed/expected ratio in the analysis. A and C nucleotides were preferred at the “1” position, while any nucleotide but G was present at the “2” position. A statistical analysis was performed [62] and only the nucleotides from “−2” to “2” were found to be significantly above background levels. After examination of the individual nucleotides at the IR-induced DNA cleavage, it was concluded that the preferred nucleotides were 5′-GGC*MH (where * is the cleavage site, M is A or C, H is any nucleotide except G).

#### 3.2.2. The End-Labelling Technique Nucleotide Preference

For the end-labelling technique data, as seen in Figure 5B, the nucleotide frequencies were determined for the 10% highest intensity cleavage sites [61]. It was found that C nucleotides were preferred at the cleavage site (position “0”), with G nucleotides also prominent. At the “−3” position, A nucleotides had the highest observed/expected frequency ratio, G nucleotides were highest at positions “−2” and “−1”, and C nucleotides were highest at the “1” position. Thus, a consensus sequence of 5′-AGGC*C can be derived from the 10% highest intensity cleavage sites with the end-labelling technique.

#### 3.2.3. LA/PS Nucleotide Preference

With the LA/PS data, as seen in Figure 5C, the nucleotide frequencies were determined for the 10% highest intensity damage sites [60]. It was found that G nucleotides were highly prominent at the damage site (position “0”) as well as position “−1”. These G nucleotides at positions “−1′ and “0” had the highest observed/expected frequency ratios by a large margin compared with other nucleotides. A consensus sequence of 5′-GG* was derived from the 10% highest intensity damage sites with the LA/PS technique.

In Figure 5, the genome-wide DNA sequence preference was well-defined with elevated observed/expected ratios only found for nucleotides at positions “−2” to “2”, while other positions had values close to 1. The end-labelling technique and LA/PS techniques had higher levels of noise in the surrounding sequence. The LA/PS technique had the highest observed/expected ratios with G nucleotides being very prominent at the damage site. For the end-labelling technique, the observed/expected ratios are less well-defined at the cleavage site compared with the other two techniques.

### 3.3. The Frequency of DNA Sequences at IR-Induced Damage Sites

The preference of DNA sequences (dinucleotides and trinucleotides) at the highest intensity IR-induced DNA damage sites was determined for the three techniques, as seen in Table 1 and Table 2. These DNA sequences were sorted by the observed/expected ratio to determine those sequences that were damaged to the greatest extent by IR. Three positions were examined for dinucleotides: “−2 −1”, “−1 0” and “0 1”, as seen in Table 1; while “−2 −1 0”, “−1 0 1” and “0 1 2” were examined for trinucleotides, as seen in Table 2. For all three techniques, G and C nucleotides predominated at the three highest intensity IR-induced DNA damage sites as measured by the observed/expected ratios.

#### 3.3.1. Dinucleotides

For position “−2 −1”, as seen in Table 1, the highest observed/expected dinucleotides were 5′-GG, GC and CC for genome-wide; 5′-AG, AT and GG for end-labelling; and 5′-CG, AG and GG for the LA/PS technique.

For position “−1 0”, as seen in Table 1, the topmost observed/expected dinucleotides were 5′-CC*, GC* and GG* for genome-wide; 5′-GC*, TC* and GA* for end-labelling; and 5′-GG* AG* and GC* for the LA/PS technique.

For position “0 1”, as seen in Table 1, the uppermost observed/expected dinucleotides were 5′-C*C, G*G and G*C for genome-wide; 5′-G*C, C*A and C*C for end-labelling; and 5′-G*G, G*C and G*A for the LA/PS technique.

#### 3.3.2. Trinucleotides

For position “−2 −1 0”, as seen in Table 2, the highest observed/expected trinucleotides were 5′-GCC*, GGC* and GGG* for genome-wide; 5′-GTC*, ATC* and AGC* for end-labelling; and 5′-AGG*, TGG*, CGG* and GGG* for the LA/PS technique.

For position “−1 0 1”, as seen in Table 2, the uppermost observed/expected trinucleotides were 5′-CC*C, GC*C and GG*C for genome-wide; 5′-TC*A, TG*C and AG*C for end-labelling; and 5′-GG*G, AG*A, GG*C and GG*T for the LA/PS technique.

For position “0 1 2”, as seen in Table 2, the topmost observed/expected trinucleotides were 5′-C*CC, G*CC and C*CT for genome-wide; 5′-C*AA, G*CG and C*AG for end-labelling; and 5′-G*GG, G*GT and G*CT for the LA/PS technique.

With the genome-wide technique, a larger number of DNA sequences could be analysed since the genome-wide data was more extensive [62]. With the genome-wide pentanucleotide analysis, the most prominent sequences were 5′-GCC*CC and 5′-GGC*CC (for positions “−2 −1 0 1 2”).

For the highest observed/expected dinucleotides and trinucleotides, it can be seen that the genome-wide technique had a high level of G and C nucleotides present in the dinucleotides and trinucleotides. The end-labelling technique had a large number of G and C nucleotides present at the damage site but more A and T nucleotides were also present. The LA/PS technique had mainly G nucleotides at the damage site with a smaller number of C, T or A nucleotides.

The preferred nucleotides at the IR-induced DNA damage site are shown from the −3 to the +2 positions. S is G or C, M is A or C, and H is any nucleotide except G [60,61,62]. Only the most highly preferred nucleotides are shown. The preferred individual nucleotides for the genome-wide data is from the top 50,000 cleavage sites with purified human DNA. The preferred individual nucleotides for the end-labelling and the LA/PS techniques was obtained from the 10% highest intensity IR-induced DNA damage sites with the Mito 15 DNA sequence. The consensus sequence from the individual nucleotide data is indicated as well as the data from complete sequence data (dinucleotide, trinucleotide and pentanucleotide).

## 4. Discussion

In this review, the sequence preference of IR-induced DNA damage was assessed using three different techniques that can detect DNA damage at nucleotide resolution, as seen in Figure 1. The genome-wide technique using Illumina next-generation DNA sequencing detects DSBs, and C nucleotides were mainly found at the DNA cleavage site [62]. The end-labelling technique mainly detects SSBs and C nucleotides were preferentially found at the DNA damage site [61]. The LA/PS technique that mainly detects base damage, detected damage predominantly at G nucleotides [60].

An expanded DNA sequence preference was also determined, and the analysis extended for ten nucleotides on either side of the DNA damage site. Two types of analysis were employed for this expanded DNA sequence preference: the individual nucleotides at the IR-induced DNA damage site; and the dinucleotides and trinucleotides present at the damage site.

### 4.1. Individual Nucleotide Analysis

With the individual nucleotide analysis, the preferred consensus sequence with the genome-wide technique was 5′-GGC*MH (where M is A or C, H is any nucleotide except G); with the end-labelling technique it was 5′-AGGC*C; and with the LA/PS technique it was 5′-GG*, as seen in Table 3. The preferred consensus sequences with the genome-wide and the end-labelling techniques were very similar, while the consensus sequence with LA/PS technique was different. This implies that a similar mechanism was operating for the IR-induced DSBs and SSBs, but a different mechanism was occurring for base damage (see below).

The genome-wide individual nucleotide sequence preference was well-defined with elevated observed/expected ratios only found for nucleotides at positions “−2” to “2”, while other positions had values close to 1. The end-labelling and LA/PS techniques had higher levels of noise in the surrounding sequence. The LA/PS technique had the highest observed/expected ratios with G nucleotides being very prominent at the damage site. For the end-labelling technique, the observed/expected ratios are less well-defined at the cleavage site compared with the other two techniques.

### 4.2. Dinucleotide and Trinucleotide Analysis

With the dinucleotide and trinucleotide analysis, the results were more complex, as seen in Table 1 and Table 2. It was observed that G and C nucleotides predominated at the highest intensity IR-induced DNA damage sites (as assessed by the observed/expected ratios) with the dinucleotide and trinucleotide analysis for the genome-wide and end-labelling techniques. The LA/PS technique had a high proportion of G nucleotides at the damage sites with the dinucleotide and trinucleotide analysis.

### 4.3. Comparison of the Individual Nucleotide Analysis with the Dinucleotide/Trinucleotide Analysis

Comparing the data derived from the individual nucleotide analysis with the dinucleotide, trinucleotide and tetranucleotide analysis, it was seen that the genome-wide technique gave similar results with the two methods. The pentanucleotides with the highest observed/expected ratios were 5′-GCC*CC and 5′-GGC*CC, which conformed to the individual nucleotide sequence preference of 5′-GGC*MH, as seen in Table 3. The genome-wide highest observed/expected data for the dinucleotides and trinucleotides were also consistent with the individual nucleotide sequence preference.

For the end-labelling data, the individual nucleotide sequence preference was 5′-AGGC*C. A number of the highest-ranking observed/expected dinucleotides and trinucleotides conformed to this sequence preference, but a number did not. The highest-ranked dinucleotide, 5′-GC*, was found in the sequence preference, whereas the highest-ranked trinucleotide, 5′-TC*A, was not found.

With the LA/PS technique, the individual nucleotide sequence preference was 5′-GG*. In general, the highest-ranking observed/expected dinucleotides and trinucleotides were consistent with this sequence preference. The highest-ranked dinucleotide, 5′-GG*, was the same as the individual nucleotide sequence preference, and the highest-ranked trinucleotide, 5′-GG*G, also contained this 5′-GG*sequence.

### 4.4. The IR-Induced Lesions Detected by the Three Techniques

#### 4.4.1. Genome-Wide Technique

The genome-wide technique is only capable of detecting DSBs. However, other DNA damage that can be converted to a DSB during processing of the IR-irradiated DNA can be detected by the technique. As part of the Illumina workflow, single-strand overhangs are enzymically polished to give blunt ends that are ligated to linker sequences. Hence, two SSBs in close proximity are likely to be converted to a DSB [67,68], especially since approximately 25–50 times as many SSBs are produced compared with DSBs [28]. In addition, abasic sites could also contribute after conversion to a strand break.

#### 4.4.2. End-Labelling Technique

The end-labelling technique detects DNA strand breaks. Again, any IR-induced DNA lesion that is converted to a strand break during processing, will also be detected by the technique. As part of the end-labelling technique, the samples are heated at 95 °C in formamide that can convert various lesions to SSBs. Thus, as well as SSBs, it is likely that abasic sites, base lesions and other sugar damage that are converted to SSBs will also be detected by the end-labelling technique. The proportion of the IR-induced DNA lesions detected by the end-labelling technique that originally derived from abasic sites, base lesions and other sugar damage, is difficult to determine in this assay.

#### 4.4.3. The LA/PS Technique

In the LA/PS assay, any IR-induced lesion that inhibits the passage of the *Taq* DNA polymerase will be detected and hence this technique can detect DSBs, SSBs, abasic sites, base damage and other lesions. The genome-wide and the end-labelling techniques mainly detect DSBs and SSBs, respectively, and the high intensity damage sites had a preference for C nucleotides at position “0” with these techniques [61,62]. The LA/PS assay can detect DSBs and SSBs but since G nucleotides were preferentially found at the DNA damage site (position “0”) and C nucleotides were low at this position, the LA/PS technique was thought to mainly detect base damage, abasic sites and other lesions, and not DSBs and SSBs [60].

Several studies with purified DNA have determined the main IR-induced base lesions. It has been observed that 30% of IR-induced base lesions were 8-oxoG, 28%—Fapy-G, 13%—5-formyl-2′-deoxyuridine, 10%—Thy-Gly, 7%—5-hydroxy-2′-deoxyuridine, 5%—5-(hydroxymethyl)-2′-deoxyuridine, 4%—Fapy-A, and 3%—8-oxoA [69]. It was calculated in another study that 42% of damaged bases were 8-oxoG, 27%—Thy-Gly, 14%—cytosine glycol, 7%—8-oxoA, 6%—Fapy-G, and 3%—Fapy-A [70]. Thus IR-induced damage at guanine bases accounted for approximately 50% of base damage, while damage at purines was found at 2–3 times the level of pyrimidines.

Fapy-G, 8-oxoG, Fapy-A and 8-oxoA are the major IR-induced degradation products of the purines [38,71,72,73]. It was observed that the methyl version of Fapy-G inhibited the extension of DNA polymerases [74,75,76] and hence it is highly likely that Fapy-G is also able to inhibit DNA polymerase. In these studies, 8-oxoG and 8-oxoA were not found to inhibit the extension of DNA polymerase. There has been a report that 8-oxoG is able to transiently inhibit DNA polymerase extension [77] and that the amplification efficiency of PCR is reduced on templates containing 8-oxoG or 8-oxoA [78]. There are no reports on the effect of Fapy-A on the extension of DNA polymerases.

During the operation of the LA/PS procedure, IR-induced lesions can be converted to DNA polymerase-blocking lesions. 8-oxoG has shown lability [79], and during the heating stages of the LA/PS procedure, it could be converted to an abasic site that would inhibit DNA polymerase extension. It has also been observed that the formation of tandem lesions involving 8-oxoG account for about 50% of 8-oxoG products [80,81] and these tandem products are highly likely to inhibit *Taq* DNA polymerase.

For the pyrimidine bases, the major IR-induced degradation products are Thy-Gly [38]. Thy-Gly lesions have been shown to be a strong block to DNA polymerase extension [82]. Other degradation products of thymine and ring saturation and oxidation products of uracil and cytosine were not found to block DNA polymerase extension.

In the LA/PS assay, there were very few high intensity sites that could be attributed to IR-induced DNA damage at T nucleotides [60]. This indicated that Thy-Gly lesions were not major sites of IR-induced DNA damage as detected by the LA/PS technique.

It is also possible that DNA polymerase is not completely blocked by an IR-induced lesion and DNA bypass may occur, especially with base lesions. Depending on the DNA sequence context, a DNA lesion may block the DNA polymerase at a frequency less than 100%.

The LA/PS technique is expected to detect abasic sites since DNA polymerase is likely to be stopped by a removed base [78,83]. As well as abasic lesions, DNA damage that can be converted to abasic sites during the LA/PS procedure (see above for 8-oxoG lability) will also be detected by the technique. Since heating above 90 °C is a step in each cycle of the linear amplification procedure, this could be a major contributor to the DNA damage detected in the LA/PS technique.

It has been observed that IR-induced base damage can be transferred along the polynucleotide chain until it reaches the site with the lowest ionisation energy [32,50,51,84,85]. Since G bases have the lowest ionisation energy in DNA, G bases accumulate more IR-induced DNA damage than expected. This process occurs with direct IR-induced base damage; however, under the IR conditions used with the LA/PS technique, direct DNA damage is only expected to make a small contribution to the overall level of IR-induced DNA damage, while indirect DNA damage is expected to overwhelmingly predominate.

### 4.5. The Mechanism of Action for IR-Induced DNA Damage

As mentioned above, under the experimental conditions employed with these techniques, the indirect effects of IR are expected to predominate while direct IR effects are expected to make a minor contribution to the observed DNA damage. The major contributor to the indirect effects of IR is the hydroxyl radical [32]. Experimental and theoretical analysis of hydroxyl radical damage to DNA indicated that the width of the minor groove is the most important determinant of the degree of hydroxyl radical damage to DNA; the wider the minor groove, the greater the extent of DNA damage. A wider minor groove would allow the hydroxyl radicals an easier path to react with the deoxyribose sugar that would eventually lead to phosphodiester strand cleavage. GC-rich DNA has a wider major groove than AT-rich DNA and hence GC bp are damaged to a greater extent than AT bp. This was confirmed with the genome-wide, end-labelling and LA/PS techniques where G and C nucleotides were preferentially damaged by IR.

The similarity of the DNA sequence preference of IR-induced DNA damage as detected by the genome-wide and end-labelling techniques implies that they are detecting a similar type of DNA damage. The genome-wide technique mainly detects DSBs and the end-labelling technique mainly detects SSBs and hence it is likely that they are caused by a similar mechanism. This mechanism is probably a hydroxyl radical that abstracts a deoxyribose sugar proton in the minor groove of DNA that leads to strand breakage.

In contrast the LA/PS technique has a different sequence preference compared with the genome-wide and end-labelling techniques. This technique is likely to be detecting base lesions and abasic sites that are derived from hydroxyl radical reactions with bases in DNA.

### 4.6. Future Work

Future experimental work could evaluate IR-induced DNA damage in aerobic vs. anaerobic conditions to investigate the effects of oxygen on the DNA sequence preference. The use of hydroxyl radical scavengers would enable the contribution of direct vs. indirect IR-induced DNA damage to be determined. Other environmental conditions could also be examined, for example, temperature, pH, and the type and intensity of radiation. A high dose of IR was used in the current experiments and future experiments with lower, clinically relevant doses would be of great interest.

The experiments were conducted with purified DNA and this is a highly artificial system and the physiological relevance is not clear. This research could be extended to examine cellular systems. It would be fascinating to observe whether the cellular environment alters the DNA sequence preference of IR-induced DNA damage. In the cellular environment, DNA is bound by proteins and cellular activity, including repair and other cellular dynamic processes, could have a major influence on the interaction between IR and DNA. For example, are the IR-induced preferential DNA damage sites with purified DNA similar to the preferential DNA damage sites in cellular DNA? Do these IR-induced preferential DNA damage sites strongly contribute to the IR-induced DNA damage response with regard to checkpoint activation, lesion repair, and genome instability? Are these preferential DNA damage sites relevant to the human diseases that are caused by defects in the DNA damage response? Future experiments with cellular systems including animals would be required to fully answer these interesting questions.

## 5. Conclusions

The three techniques that were examined in this review could detect a variety of IR-induced DNA lesions, with the Illumina next-generation genome-wide sequencing technique mainly detecting DSBs, the end-labelling procedure mainly detecting SSB, and the LA/PS assay mainly detecting base damage. It should be noted that there are major methodological differences between the three techniques, and this should be taken into account when comparing the data from the three systems. The human DNA utilised for the genome-wide experiments was more complex than the plasmid DNA used for the end-labelling and LA/PS experiments, and hence a slightly higher radiation dose was required to achieve optimal results. This should be considered when comparing the three procedures. The genome-wide and the end-labelling techniques had a similar IR-induced DNA sequence preference and found C nucleotides at the damage site (position “0”) with mainly G and C nucleotides in the surrounding sequence; while the LA/PS technique revealed G nucleotides at the damage site (position “0”) with mainly G nucleotides in the surrounding sequence. The similarity of the results with the genome-wide and the end-labelling techniques implied that a similar mechanism was operating for the production of IR-induced DSBs and SSBs, with hydroxyl radicals reacting with deoxyribose sugars in the minor groove of DNA to give rise to strand breakage. The G and C nucleotide preference is probably due to the wider minor groove of GC bp compared with AT bp. With the LA/PS technique, the sequence preference is for G nucleotides and is likely to be detecting base lesions and abasic sites that are derived from hydroxyl radical reactions with bases in DNA. Thy-Gly lesions were not major sites of IR-induced DNA damage with the LA/PS technique. Fapy-G and 8-oxoG are possibly the main IR-induced lesions detected by the LA/PS technique. In summary, these three techniques have provided deeper insight into the sequence preference of IR-induced DNA damage.

## Figures and Tables

**Table 1:** The observed/expected ratios for γ radiation-induced DNA damage at dinucleotides.

**A.**					
**Genome-Wide**		**End-Labelling**		**LA/PS**	
**“** **−2 −1”**	**Obs/Exp**	**“** **−2 −1”**	**Obs/Exp**	**“** **−2 −1”**	**Obs/Exp**
GG	2.4	AG	2.39	CG	4.16
GC	2.4	AT	1.89	AG	2.91
CC	1.9	GG	1.55	GG	2.06
AG	1.5	GC	1.50	AA	1.59
CA	1.2	GT	1.43	TG	1.41
CT	1.2	CG	1.30	GA	1.10
GA	1.1	CT	1.11	CC	0.99
TG	1.1	TA	1.02	CA	0.94
GT	0.9	CA	0.91	GT	0.90
AC	0.9	AC	0.78	TA	0.73
TC	0.9	GA	0.73	AT	0.68
AA	0.6	TG	0.71	CT	0.55
CG	0.5	TT	0.59	TC	0.26
TT	0.5	CC	0.21	GC	0.00
AT	0.4	AA	0.00	AC	0.00
TA	0.3	TC	0.00	TT	0.00
**B.**					
**Genome-Wide**		**End-Labelling**		**LA/PS**	
**“−1 0”**	**Obs/Exp**	**“−1 0”**	**Obs/Exp**	**“−1 0”**	**Obs/Exp**
CC*	2.5	GC*	3.99	GG*	7.79
GC*	2.3	TC*	2.44	AG*	3.89
GG*	2.2	GA*	1.95	GC*	1.67
CT*	1.5	GG*	1.74	CG*	1.49
AG*	1.3	TG*	1.66	GT*	1.29
TG*	1.2	AC*	1.17	GA*	1.05
CA*	1.1	AG*	1.09	TT*	1.00
AC*	1.1	CC*	0.85	TG*	0.90
TC*	1.0	TA*	0.83	AC*	0.69
GT*	0.8	CG*	0.83	AT*	0.65
GA*	0.8	AA*	0.29	CC*	0.34
CG*	0.6	CA*	0.23	CT*	0.27
TT*	0.5	CT*	0.22	TC*	0.24
AA*	0.5	GT*	0.00	AA*	0.00
AT*	0.4	AT*	0.00	CA*	0.00
TA*	0.3	TT*	0.00	TA*	0.00
**C.**					
**Genome-Wide**		**End-Labelling**		**LA/PS**	
**“0 1”**	**Obs/Exp**	**“0 1”**	**Obs/Exp**	**“0 1”**	**Obs/Exp**
C*C	2.3	G*C	3.33	G*G	5.56
G*G	1.6	C*A	3.25	G*C	2.78
G*C	1.6	C*C	2.34	G*A	2.63
C*A	1.4	A*G	1.74	G*T	2.58
C*T	1.4	G*G	1.52	C*T	1.08
T*C	1.2	G*T	1.20	T*A	1.05
G*A	1.0	A*T	0.81	T*G	0.90
G*T	1.0	C*T	0.67	C*A	0.68
T*G	0.9	A*C	0.60	C*G	0.50
T*T	0.9	G*A	0.49	T*T	0.50
A*C	0.9	T*G	0.24	T*C	0.48
A*G	0.8	A*A	0.00	A*A	0.38
A*A	0.7	T*A	0.00	A*T	0.33
A*T	0.6	T*T	0.00	A*C	0.00
T*A	0.5	T*C	0.00	A*G	0.00
C*G	0.3	C*G	0.00	C*C	0.00

The IR-induced observed/expected (Obs/Exp) ratios are listed in descending order for the sixteen possible dinucleotides [60,61,62]. The observed/expected ratios were listed: (**A**) for position “−2 −1”, (**B**) for “−1 0” and (**C**) for “0 & 1”, with “0” corresponding to the damage/cleavage site. The * indicates the damage site and the sequences are written 5′ to 3′. For the genome-wide data, the observed/expected ratios were calculated as a ratio of dinucleotide frequency in the top 50,000 sites compared with the overall dinucleotide frequency in the human genome. With the end-labelling procedure, the data was obtained with the Mito 15 DNA sequence. The observed/expected ratios were calculated from the 10% highest intensity IR-induced DNA damage sites. The data for the LA/PS technique was calculated from the 10% highest intensity IR-induced DNA damage sites with the Mito 15 DNA sequence. A statistical analysis was not performed for the data in this table.

**Table 2:** The observed/expected ratios for γ radiation-induced DNA damage at trinucleotides.

**A.**					
**Genome-Wide**		**End-Labelling**		**LA/PS**	
**“−2 −1 0”**	**Obs/Exp**	**“−2 −1 0”**	**Obs/Exp**	**“−2 −1 0”**	**Obs/Exp**
GCC*	4.5	GTC*	4.84	AGG*	7.21
GGC*	4.0	ATC*	4.84	TGG*	7.21
GGG*	3.6	AGC*	4.15	CGG*	6.41
CCC*	3.1	TGC*	3.87	GGG*	6.41
AGC*	2.7	CTC*	3.69	CAG*	4.81
GCT*	2.6	CTG*	3.63	CCG*	4.81
CAG*	2.5	GAC*	3.23	GAG*	4.81
CTG*	2.4	GCC*	2.77	AAG*	3.85
AGG*	2.4	GCA*	2.42	CGT*	3.85
GAG*	2.0	TTC*	2.42	AGA*	3.20
**B.**					
**Genome-Wide**		**End-Labelling**		**LA/PS**	
**“−1 0 1”**	**Obs/Exp**	**“−1 0 1”**	**Obs/Exp**	**“−1 0 1”**	**Obs/Exp**
CC*C	5.1	TC*A	7.45	GG*G	9.61
GC*C	4.4	TG*C	5.81	AG*A	6.41
GG*C	3.2	AG*C	5.54	GG*C	6.41
GG*G	3.0	AC*A	4.84	GG*T	6.41
CT*C	2.8	TC*C	4.31	AG*G	4.81
GC*A	2.4	GC*T	4.15	TG*G	4.81
CC*A	2.3	GA*C	3.23	GG*A	3.85
CC*T	2.2	GC*C	2.77	AG*T	3.20
AG*C	2.0	GC*A	2.42	AG*C	2.75
TC*C	2.0	CG*G	2.42	GC*A	2.75
**C.**					
**Genome-Wide**		**End-Labelling**		**LA/PS**	
**“0 1 2”**	**Obs/Exp**	**“0 1 2”**	**Obs/Exp**	**“0 1 2”**	**Obs/Exp**
C*CC	3.5	C*AA	6.92	G*GG	6.41
G*CC	2.5	G*CG	4.84	G*GT	6.41
C*CT	2.4	C*AG	4.84	G*CT	5.77
C*CA	2.3	C*CT	4.52	G*AC	4.81
C*AG	2.1	G*CT	4.15	G*AG	4.81
G*GG	2.1	C*AC	3.99	G*TA	3.85
T*CC	2.1	A*CG	3.63	G*GC	3.20
C*TG	2.0	C*AT	3.52	G*CA	2.75
G*GC	2.0	A*TC	3.23	C*AG	2.40
C*TC	1.8	C*TT	3.23	C*GA	2.40

The IR-induced observed/expected (Obs/Exp) ratios are listed in descending order for the ten highest ratios for trinucleotides [60,61,62]. The observed/expected ratios were listed: (**A**) for position “−2 −1 0”, (**B**) for “−1 0 1” and (**C**) for “0 1 2”, with “0” corresponding to the damage/cleavage site. The * indicates the damage site and the sequences are written 5′ to 3′. For the genome-wide data, the observed/expected ratios were calculated as a ratio of trinucleotide frequency in the top 50,000 sites compared with the overall trinucleotide frequency in the human genome. With the end-labelling procedure, the data was obtained with the Mito 15 DNA sequence. The observed/expected ratios were calculated from the 10% highest intensity IR-induced DNA damage sites. The data for the LA/PS technique was calculated from the 10% highest intensity IR-induced DNA damage sites with the Mito 15 DNA sequence. A statistical analysis was not performed for the data in this table.

**Table 3:** The preferred nucleotides at the DNA damage site induced by IR.

Study	Type of DNA Damage	Preferred Individual Nucleotides						Consen Sus Sequence from the Individual Nucleotide Data	From Complete Sequence Data
Position		−3	−2	−1	0 *	1	2		
Genome-wide purified human DNA (50 k)	DSB	-	G	G	C	C > A	H	5′-GGC*MH	5′-GGC*CC 5′-GCC*CC
End-labelled DNA	SSB	A	G	G	C	C	-	5′-AGGC*C	5′-GC*
LA/PS DNA	base	-	-	G	G	-	-	5′-GG*	5′-GG*G

The preferred nucleotides at the IR-induced DNA damage site are shown from the −3 to the +2 positions. S is G or C, M is A or C, and H is any nucleotide except G [60,61,62]. Only the most highly preferred nucleotides are shown. The preferred individual nucleotides for the genome-wide data is from the top 50,000 cleavage sites with purified human DNA. The preferred individual nucleotides for the end-labelling and the LA/PS techniques were obtained from the 10% highest intensity IR-induced DNA damage sites with the Mito 15 DNA sequence. The consensus sequence from the individual nucleotide data is indicated as well as the data from complete sequence data (dinucleotide, trinucleotide and pentanucleotide).

## Abbreviations

bp	base pair
CE-LIF	Capillary electrophoresis with laser-induced fluorescence detection
DSB	Double-strand breaks
Fapy-A	4,6-diamino-5-formamidopyrimidine
Fapy-G	2,6-diamino-4-hydroxy-5-formamidopyrimidine
LA/PS	Linear amplification/polymerase stop
IR	Ionising radiation
8-oxoA	8-oxo-7,8-dihydro-2′-deoxyadenosine
8-oxoG	8-oxo-7,8-dihydroguanine
SSB	Single-strand breaks
Thy-Gly	cis- and trans-diasteromers of 5,6-dihydroxy-5,6-dihydrothymine
H	any nucleotide except G
M	A or C
N	any nucleotide
S	G or C
W	A or T
